# COVID-19 and 5G conspiracy theories: long term observation of a digital wildfire

**DOI:** 10.1007/s41060-022-00322-3

**Published:** 2022-05-27

**Authors:** Johannes Langguth, Petra Filkuková, Stefan Brenner, Daniel Thilo Schroeder, Konstantin Pogorelov

**Affiliations:** 1 Department of High Performance Computing, Simula Research Laboratory, Oslo, Norway; 2 Department Data Science and Analytics, BI Norwegian Business School, Oslo, Norway; 3 Department of Psychology, Inland Norway University of Applied Sciences, Lillehammer, Norway; 4 Department of Information and Communication, Stuttgart Media University, Stuttgart, Germany

**Keywords:** Misinformation, Digital wildfire, Twitter, COVID-19, Conspiracy theories

## Abstract

The COVID-19 pandemic has severely affected the lives of people worldwide, and consequently, it has dominated world news since March 2020. Thus, it is no surprise that it has also been the topic of a massive amount of misinformation, which was most likely amplified by the fact that many details about the virus were not known at the start of the pandemic. While a large amount of this misinformation was harmless, some narratives spread quickly and had a dramatic real-world effect. Such events are called digital wildfires. In this paper we study a specific digital wildfire: the idea that the COVID-19 outbreak is somehow connected to the introduction of 5G wireless technology, which caused real-world harm in April 2020 and beyond. By analyzing early social media contents we investigate the origin of this digital wildfire and the developments that lead to its wide spread. We show how the initial idea was derived from existing opposition to wireless networks, how videos rather than tweets played a crucial role in its propagation, and how commercial interests can partially explain the wide distribution of this particular piece of misinformation. We then illustrate how the initial events in the UK were echoed several months later in different countries around the world.

## Introduction

On January 21, 2020, the first message which linked the COVID-19 outbreak in Wuhan, China, to 5G wireless technology appeared on Twitter, stating: *“China is 5G now & working toward 6G. Wireless radiation is an immunosuppressor. Conincidence?”*. The tweet got little reaction, but in the following days, a series of similar tweets appeared. Their number grew steadily and about ten weeks later, in early April, a series of arson attacks hit wireless network equipment, mostly cell towers, in the UK as well as in Ireland, the Netherlands, Cyprus, and New Zealand. With these attacks, the misinformation became a *digital wildfire*.

Digital wildfires, which are defined as fast-spreading and inaccurate, counterfactual, or intentionally misleading pieces of information that quickly permeate public consciousness and have serious real-world implications, have been placed among the top global risks in the twenty-first century by the World Economic Forum [27]. While a sheer endless amount of misinformation exists on the Internet, only a small fraction of it spreads far and affects people to such a degree that they commit harmful acts in the real world. The alleged connection between 5G and COVID-19 differed little from other counterfactual statements which are common on the internet. However, it had a far greater effect. Similar events have been observed before, e.g., misinformation linked to mob violence in India [11] where several random people were killed after rumors about child abduction appeared on WhatsApp and were shared widely. Here, a single message in a social network was spread among a massive number of users, which was relatively easy to track afterward. On the other hand, the perceived link between 5G and COVID-19 was not caused by a single message in a social network. Instead, the digital wildfire consisted of many messages on Twitter and other social networks, which combined into a single misinformation event. We call such events *complex digital wildfires*.

Clearly, investigating complex digital wildfires is far more challenging than investigating ordinary (i.e., simple) digital wildfires. In this paper, we perform such an analysis for misinformation that links 5G and COVID-19 in order to understand its origins and the factors that contributed to its spread. We search through a massive set of COVID-19-related messages from Twitter that were archived as early as January 2020, thereby finding the first tweets that mention a connection between 5G and COVID-19. Based on a manually labeled dataset, we train a machine learning classifier to distinguish between tweets that insinuate a connection between unrelated terms and those that only mention both without claiming a connection. We chart the number of such tweets over time and analyze the geographical distribution. Our goal is to investigate six fundamental questions:

1. How did the 5G-COVID misinformation event start and where did it come from in January 2020?
2. How did it grow from relative obscurity to a widely discussed topic in late March 2020?
3. What is the connection to the serious real-world consequences observed in April 2020?
4. How did the wildfire develop around the globe between April 2020 and November 2021?
5. Which general observations can we make from the structure of the specific event?
6. What lessons and policy recommendations can we draw from these observations?

In the remainder of the paper, we first discuss the real-world events associated with the wildfire and investigate its origins using qualitative methods in Sect. 2. We then extend the analysis to the main body of misinformation in Sect. 3. By investigating a large number of Twitter messages, we lay the groundwork for quantitative analysis via machine learning methods, the results of which are presented in Sect. 4. In Sect. 5, we discuss the interplay between the spread of this misinformation on Twitter and on video platforms such as YouTube, and we investigate the motivations for spreading such misinformation in Sect. 6. We finish the paper by discussing related work in Sect. 7 and present our conclusions in Sect. 8.

## The initial 5G-COVID digital wildfire

Different misinformation narratives that imply a causal link between 5G and COVID-19 have spread on social media, some of which are contradictory. Some suggest that the electromagnetic radiation emitted by 5G equipment causes COVID-19, while others state that 5G harms people directly and that COVID-19 is used to hide this fact. We provide a more detailed description of these narratives in Sect. 3.4, but throughout this paper, we consider all such misinformation narratives together.

We use the term *5G-COVID misinformation event* to refer to the entirety of all communication that imply a causal link between 5G and COVID-19, i.e., all Twitter messages, Facebook posts, news articles, and videos. In addition, we consider all real-world events such as arson attacks that are presumably a direct consequence of this misinformation to be part of the event. Since it is essentially impossible to obtain an incontrovertible proof of causality between misinformation and real-world harm, we include all events where such a causality is highly likely.

The initial event happened almost exclusively on social media from January to March 2020, with the international press taking essentially no notice of it (see Sect. 3.5). Between March 25 and April 2, following the release of YouTube videos promoting 5G-COVID misinformation, the daily number of tweets dealing with the topic grew fourfold. Immediately thereafter, a series of arson attacks on mobile network infrastructure in the UK and other countries began and continued for several weeks. The attacks got immediate and widespread attention by established media in the UK and in many other countries.

On April 5, YouTube announced a ban on videos that spread 5G-COVID misinformation and removed most such videos [38]. On April 22, Twitter announced that it would ban tweets and users that call for attacks on 5G infrastructure [15]. The company later added fact checking links to tweets mentioning 5G and COVID-19. Since then, the number of tweets on the topic has declined, but the topic returned periodically in different places around the world throughout 2021.

### Attacks on telecommunication technicians and infrastructure

Arson attacks on telecommunications equipment have happened before 2020, for example, in Germany between 2013 and late 2019 [7, 18, 19], which may have been motivated by opposition to wireless communications. However, we only count events that happened during the COVID-19 pandemic as part of the 5G-COVID digital wildfire.

These events include arson attacks on infrastructure as well as attacks on or harassment of technicians. On the weekend of Friday, April 3, 2020, at least ten arson attacks happened in the UK, New Zealand, and the Netherlands, and more than 20 additional attacks happened one week later, predominantly in the UK. A series of six similar cases followed in Canada two weeks later. The total rose to 77 in the UK alone by May 7 [76] and to 121 by July 2 [22]. Another four arson attacks happened on July 3 in Cyprus [50].

Meanwhile, technicians who were perceived to be installing 5G infrastructure were harassed or attacked, with 273 reported incidents in the UK [22], most of them minor. A major event in this regard was the kidnapping of eight technicians in Peru on June 10 [1]. Just like in the UK attacks, the perpetrators clearly stated the perceived threat of 5G as the motivation for their actions. These statements, combined with the fact that the incidents happened shortly after the 5G-COVID misinformation attained wider recognition, imply a causal relationship between the two, which justifies the use of the term *digital wildfire*.

While the initial series of attacks ended in July 2020, several additional attacks have occurred thereafter, including one in South Africa in early 2021 [2] and a suspected case of arson in Canada by the end of March 2021 [51]. A list of real-world events connected to the 5G-COVID digital wildfire can be found in Table 3 in Appendix.

**Fig. 1:**
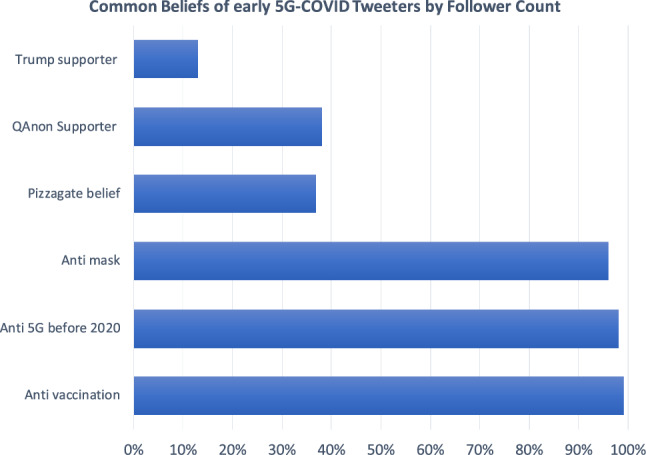
Ideas expressed by the 37 accounts that were the first to spread 5G-COVID misinformation in January

### Origin of the 5G-COVID misinformation

Different origins of the 5G-COVID idea have been proposed in the media [28, 77]. A *Wired* article [69] identified a Belgian newspaper article from January 22, 2020, as the source of the 5G-COVID misinformation, but our Twitter data collection, which is discussed in detail in Sect. 4, shows that the first tweets that connect 5G and COVID-19 appeared on January 21, 2020. It is not certain whether the event originated on Twitter, but there is substantial evidence that the idea grew out of existing opposition to 5G technology [3, 31].

During the remainder of January, 698 tweets and 1,089 retweets that mention 5G and COVID-19 appeared, of which 23 tweets and 127 retweets were classified as promoting the 5G-COVID idea by our automated classifier (see Sect. 4.1). In addition, several videos proposing similar ideas appeared on YouTube. Some of those videos belong to channels that also promote other misinformation. Several such channels have a comparatively large number of subscribers (between 50,000 and 300,000). The daily number of tweets on that topic grew slowly during January and most of February 2020.

On March 11, 2020, the WHO declared COVID-19 a global pandemic [23]. In the following weeks, along with the growing number of infections in Europe and the USA, media attention to COVID-19 pandemic grew sharply, peaking between March 20 and March 25. Shortly thereafter, a series of additional videos from UK-based sources promoting 5G-COVID narratives appeared on YouTube and other video platforms and were shared widely on Twitter.

## Qualitative analysis

To understand the 5G-COVID digital wildfire, we combine qualitative and quantitative approaches. We first describe the qualitative results since they constitute the basis for the quantitative analysis.

**Table 1:** Overview over the tweet sets used in this paper

Class	COVID-19 Tweets	5G Tweets	5G-COVID Misinformation	Other Conspiracy Tweets
Superset	Twitter	COVID-19	5G Tweets	5G Tweets
Obtained by	Twitter API	Text search	MLP classification	MLP classification
Pure misinformation	No	No	Yes	Yes
Total (re)tweets	2,186,199,057	1,181,332	134,732	74,013
Total tweets	464,702,084	398,190	20,436	20,701
Location (re)tweets	1,124,401,420	597,902	68,245	38,122
Location tweets	264,749,471	202,471	10,298	10,576

The COVID-19 set is scraped from Twitter, 5G is obtained from COVID-19, and the other two are selected from 5G by a neural network classifier. Except for false positives, they contain only misinformation tweets

### Analysis of early twitter posts

In order to understand where the idea of the 5G-COVID connection came from, we investigate the earliest tweets that mention it. A total of 104 COVID-19-related tweets by 75 accounts were made between January 21 and January 25, 2020. Among those, we identified 38 accounts that were insinuating some kind of 5G-COVID connection. 28 of these presented themselves as belonging to individual persons. One account belonged to a satirical website. We removed this account from the sample. Nineteen accounts reported their location as USA, 9 as UK, AUS, NZ, or CAN, and 9 reported other European or Asian countries as their location. We classified these 37 accounts with respect to six features, the first three features being support for Donald Trump, the QAnon conspiracy theory, and the Pizzagate and Wayfair conspiracy theories [49, 66]. We combined the Pizzagate and Wayfair conspiracies since they are very similar, with the latter being located in Europe.

The second set of features holds statements against vaccinations, against wearing face masks during the COVID-19 pandemic, and statements on health risks of 5G prior to 2020. Figure 1 shows the results, scaled by the number of followers of the accounts.

Since the first three features are political in nature, it is to be expected that they have less global appeal than the latter three which are health related. Among the US accounts, 11 out of 19 showed support for Trump (same for QAnon), while 27 out of 37 accounts voiced anti-vaccination and anti-5G opinions. Note that anti-vaccination refers to vaccines against diseases other than COVID-19. Additionally, such accounts had far more followers, as shown in Fig. 1. Thus, there is a strong indication that the very early conversation on the 5G-COVID connection was dominated by people who oppose 5G rather than members of the political right.

### Creation of the initial dataset

We perform an extensive analysis of 5G- and COVID-19-related Twitter data in order to better understand the misinformation event. Thus, the first step was to obtain this data from Twitter. The data collection was performed using a custom framework [62] to access the Twitter search API to collect a large number of statuses (i.e., *tweets*, *retweets*, *quotes*, and *replies*) containing COVID-19 related keywords such as *Corona*, *Coronavirus*, and *COVID*. The data collection started on January 17, 2020, and ended on November 30, 2021. A list of the keywords is given in appendix. The resulting set of tweets forms the initial set which we refer to as the COVID-19 set of tweets.

Next, we filtered for those that mention *“ 5G ”* and *“ 5g ”*. We did not remove the whitespaces because doing so produced too many false positives completely unrelated to 5G. We also included alternative spellings such as *“ 5-G ”*, although the number of tweets containing these is negligible. We refer to the resulting set of tweets as the 5G set. It is a subset of the COVID set and a superset of the misinformation sets that we discuss in Sect. 4. We also create a subset of each set by filtering out retweets. The total counts for these sets are listed in Table 1.

Our goal is to perform a quantitative analysis of all available 5G-COVID tweets, which will be presented in Sect. 4. However, even without retweets, our initial dataset contained 398,190 5G tweets, all of which are potential 5G-COVID, i.e., misinformation tweets. Analyzing all these tweets is impossible without an automated system, but in order to train a machine learning classifier, we need manually labeled tweets.

### Manually labeled dataset

As part of this investigation, the authors read and labeled more than 10,000 candidate tweets. For each tweet, we denoted whether it was promoting a 5G-COVID idea or not. We also included a third category for tweets that promote other conspiracy theories, such as claims that *“COVID is a hoax”* or similar ideas. The dataset was also made available to other researchers [56, 57]. With the help of this dataset, it was possible to develop an automated detection method described in Sect. 4.1. Furthermore, performing the manual labeling of such a large number of tweets gave the authors valuable insights into the common ideas and specific underlying misinformation narratives used in 5G-COVID tweets. This allowed us to perform an extensive qualitative analysis of these narratives.

### Taxonomy of misinformation narratives

The creation of the dataset allowed us to qualitatively understand the ideas promoted in 5G-COVID misinformation tweets. We found that a multitude of different underlying narratives exist that suggest the existence of a connection between 5G wireless technology and the COVID-19 pandemic, many of which are mutually exclusive. However, they all share the belief that 5G is harmful in some way and that the technology should not be installed, or existing installations should be dismantled.

Our goal is to provide a qualitative overview over the main narratives of 5G-COVID misinformation since they all constitute a possible basis for attacks on mobile communication infrastructure. However, due to the nature of the misinformation, which contains a wide range of wildly contrafactual statements and highly unlikely conspiracy theories, it is neither feasible nor desirable to perform an exhaustive quantitative analysis here. We found the following main narratives:

1. The *Immunosuppressor* narrative suggests that radiation from 5G antennas weakens the immune system, thereby making people highly susceptible to an otherwise harmless virus.
2. The *Cover-up* narrative states that radiation of 5G equipment is lethal, and that deaths attributed to the COVID-19 pandemic are actually caused by 5G. The pandemic is alleged to have been invented to hide this.
3. The *Mind Control Conspiracy* assumes that 5G allows some form of mind control and that the COVID-19 or a vaccine against it plants a receiver within humans.
4. Finally, there are some truly *Esoteric Conspiracy Theories* that see 5G and COVID-19 as means to prevent humans from reaching their full potential or higher selves.

While these ideas may sound outlandish and contradict all established scientific consensus, it is clear that belief in these ideas could drive people to violent action since they typically assert that 5G by its very nature is life- or even existence-threatening in some way.

Furthermore, in addition to the opposition against 5G in itself, the tweets also show another angle. In the context of 5G, the use of equipment manufactured by the Chinese companies Huawei and ZTE has been widely discussed as a potential cybersecurity risk in Western countries. As a result, some countries have excluded Chinese vendors from supplying 5G infrastructure. In the US, the Trump administration passed the *Secure 5G and Beyond Act* [34] which was introduced on March 27, 2019 and signed on March 23, 2020. The UK later followed suit [21], reversing an earlier decision from January 28, 2020 [75]. Consequently, part of the opposition to 5G in the US stems from an anti-China sentiment of the political right, rather than opposition to the technology of 5G. One website from that spectrum [6] tweeted on April 17, 2020: “If you like Corona in your country, you’ll love Huawei in your home”. Here, the alleged connection between 5G and COVID-19 is simply based on the fact that both come from China. The narrative is essentially that COVID-19 proves that the Chinese leadership is not trustworthy, and consequently Chinese technology should not be used. An early tweet from January 26, 2020, by a pro-Republican account states: “We can end the adoption of Chinese 5G spy technology in the West by convincing people it causes #coronarvirus outbreaks.” It should be noted that this account has only 29 followers and that the tweet generated no reactions. Therefore, it is unlikely to have led to the implementation of such a strategy.

**Fig. 2:**
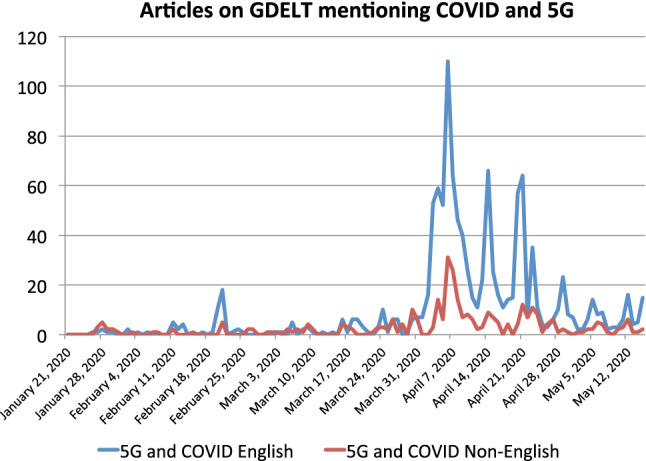
Weekly number of articles related to 5G and COVID-19 during the period of observation

### Tracking the misinformation event on GDELT

Before the attacks in April 2020, 5G-COVID misinformation was not known to a wider audience, and it appears that it was essentially invisible in traditional news media. We test this assumption by querying the *Global Database of Events, Language, and Tone* (GDELT) [47], a database of news events captured worldwide in close to real time.

We searched the records from January to May 2020 using a custom high-performance mining system [58]. The GDELT database only contains the titles of news articles, not the text body. Therefore, we used an aggressive text mining approach. We converted every news publication record into plain text format, stripping out all non-numerical and non-alphabetical symbols. Next, we truncated all excessive word separators and performed a case-insensitive search for two sets of keywords, the first being the list of COVID-19 keywords detailed in Appendix, and the second being the various spellings of 5G. As a result, using all available keyword sources including full URL links and titles where they were available, we managed to get a relatively high number of relevant news articles, i.e., event mentions, as shown in Fig. 2.

Among the early articles mentioning COVID-19 and 5G, most discuss the impact of the pandemic on 5G rollout or equipment production. However, one article does not only mention but promote the idea of a direct connection between 5G and COVID-19 [73]. It was published on February 20, 2020. We found no other such article. Table 2 in Appendix lists the articles that appeared in the seven days starting February 20, i.e., following the conspiracy-supporting news article. Out of 32 articles found via a keyword search for the selected week, only the aforementioned article discussed the 5G-COVID conspiracy. All other articles are focused on the impact of COVID-19 on 5G technology development and deployment. Furthermore, a set of 14 out of 32 articles (which all have a different URL) are copies of the same article published by Reuters.

Thus, we note that traditional media played essentially no role in the formation of this digital wildfire. They only became involved when real-world events occurred, which is clearly visible in Fig. 2. The large increase in articles in April 2020 is due to reporting about the attacks on the telecommunications equipment, i.e., the events of the wildfire itself. As a result, when considering wildfire prediction and preparedness, the analysis should clearly focus on social media.

## Quantitative analysis on twitter

In Sect. 3, we described the creation of the COVID-19 Twitter dataset, the search for 5G tweets inside this set, and the manual labeling of 5G-COVID tweets. In this section, we describe the development of an automated classifier from this dataset and the analysis of the results we obtained using this classifier.

**Fig. 3:**
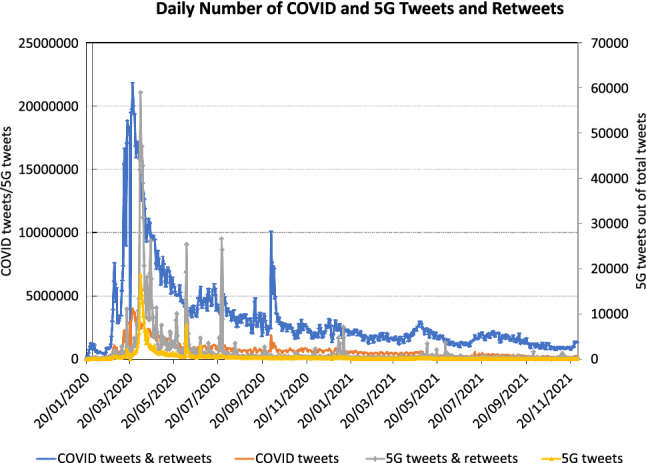
Daily number of COVID-19 (primary axis) and 5G (secondary axis) tweets and retweets in the dataset

### Automated misinformation detection

Naturally, simply observing the number of times 5G is mentioned on Twitter is not sufficient for a detailed analysis. On the other hand, manually evaluating almost 400,000 tweets is not feasible. While it would be possible to sample 400 tweets to obtain significance at $p=0.05$, doing so for each of the 680 days in the observed time span is not.

Thus, we instead opted for developing a machine learning based classifier to understand which fraction of the tweets that mention 5G and COVID-19 actually insinuate a causal relationship and thus spread misinformation. Initial sampling suggested that there are several keywords that are typically used in misinformation tweets (e.g., “immunosupressor”), but other tweets that mock or parody such tweets make use of these words as well. Thus, even for human readers, it is not always easy to discern the meaning of a tweet.

As mentioned in Sect. 3.2, we previously created a dataset of 10,000 manually annotated tweets [56, 57] that served as a training set for the automated classifier. Tweets were classified as either *i) spreading 5G-COVID misinformation*, *ii) spreading other conspiracy theories*, or *iii) not spreading conspiracy theories*. Significant effort was made to develop and test automatic classifiers for that dataset [63].

Note that *other conspiracy theories* refers to both older conspiracy theories (or myths) that have been classified previously [12], as well other misinformation with similar characteristics that appeared in the context of COVID-19, such as that the virus does not exist or was released intentionally, or that the objective of vaccines is mind control. Note however that since we only classify tweets that mention 5G, this does not constitute a general investigation of such misinformation.

We chose a multilayer perceptron (MLP) as the baseline classifier [61]. MLPs are designed to approximate any continuous function and can solve problems that are not linearly separable, which are the major use cases of pattern classification, recognition, prediction, and approximation with nonlinear mapping between input and corresponding output vectors. Furthermore, MLPs are very fast and efficient to compute, which is especially important in our case since we are dealing with a large dataset. During the initial experiments, we tried other established machine learning classifiers, such as random tree, random forest, and support vector machine. However, the resulting classification performance was low, probably due to too complex relations between classifier input features. Thus, we opted to keep focusing on MLPs. Furthermore, the size of the annotated dataset was deemed to be insufficient for more advanced deep learning (DL)-based approaches.

Due to the difficulty of the task, even MLP as the best classifier produces a significant number of false positives and negatives. However, the attained accuracy is more than sufficient to quantify the number of misinformation tweets on a daily basis. In total, 134,732 tweets and retweets were flagged as 5G-COVID misinformation, and another 74,013 were deemed to promote some other conspiracy theory, as shown in Table 1. Interestingly, the 5G tweets received fewer retweets than the average COVID-19 tweet, but among the 5G tweets, those that spread 5G-COVID misinformation received far more retweets, and tweets containing other conspiracy theories received an average number.

### Development over time

Our primary goal is to understand how the 5G-COVID misinformation developed over time. To that end, we plot the number of tweets in the dataset for each day during the time of observation. As mentioned above, we keep two counts: the first being the total number of Twitter statuses in each class, while the second does not contain retweets. The numbers are given in Fig. 3.

The number of COVID-19 tweets peak on March 24, 2020, and retweets peak one day later. Thereafter, the number of tweets quickly declines, reaching an almost steady state in late 2020 and throughout 2021. Note that the secondary peak on October 2 is caused by the COVID-19 infection of Donald Trump. It is therefore not related to 5G-COVID misinformation.

For the 5G tweets, the peak is reached on April 4, 2020, i.e., immediately after the attacks on 5G towers. There is a secondary peak on June 7, 2020, which coincides with tower attacks in New Zealand. A third peak in July 2020 consists predominantly of retweets. A smaller fourth peak at the beginning of 2021 coincides with attacks in South Africa.

**Fig. 4:**
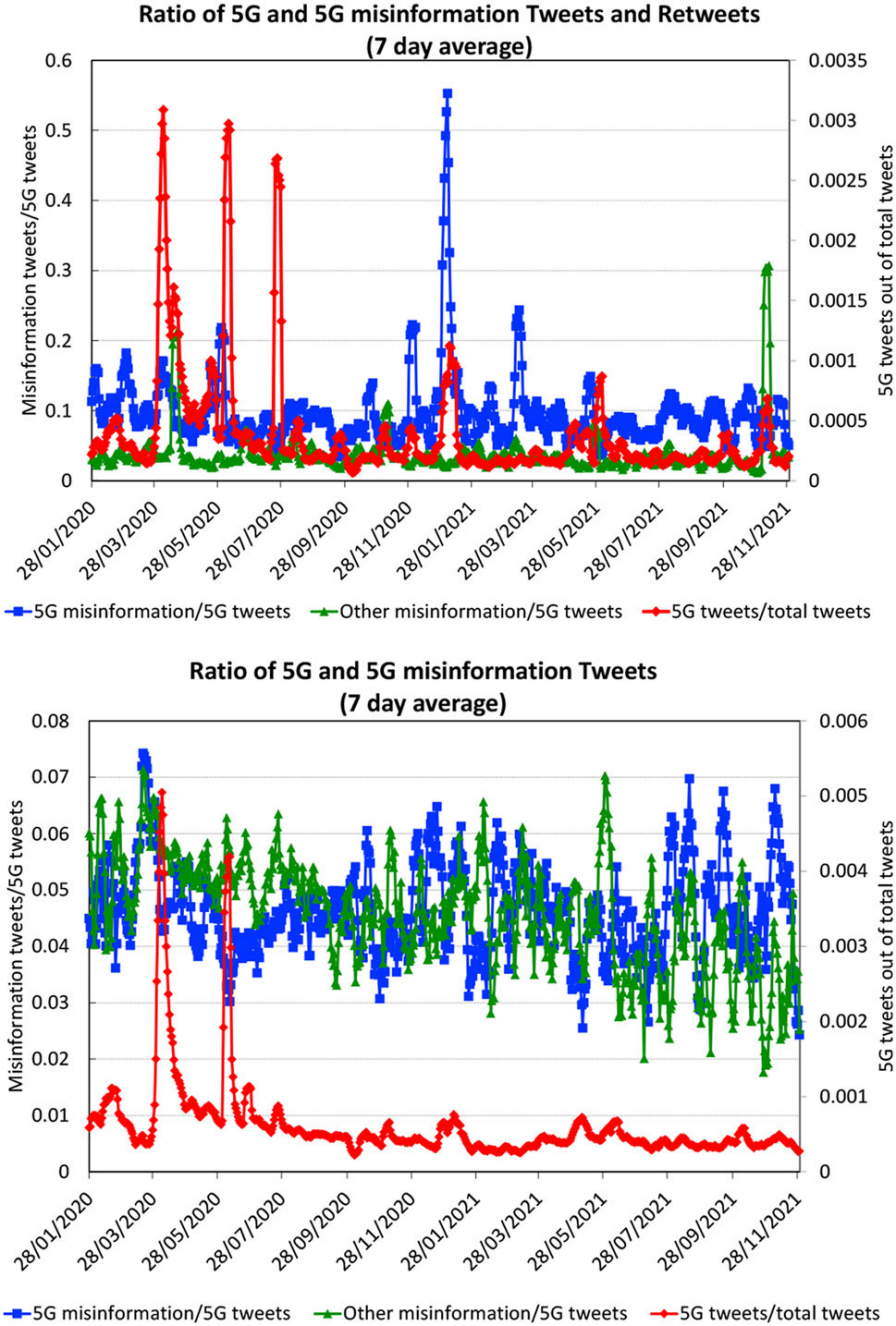
Ratio of misinformation tweets out of all 5G tweets, showing both 5G-COVID misinformation and other conspiracy theories. On the right axis in red, we also denote the ratio of 5G tweets among all COVID-19 tweets. Each data point is the average of seven daily values around the date

**Fig. 5:**
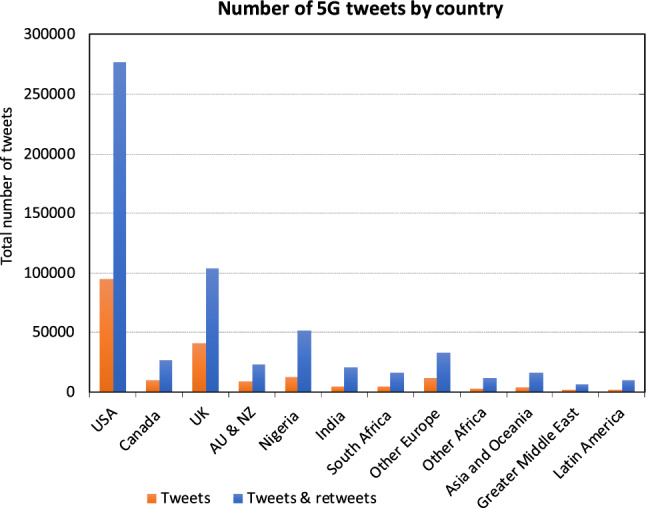
Number of tweets and retweets by country

In Fig. 4, we show the results of the machine learning classification as ratios of 5G and other misinformation tweets out of all 5G tweets. We also show the ratio between 5G tweets and all COVID-19 tweets on the secondary axis. This curve shows the same peaks as the total numbers discussed above.

The ratio of 5G misinformation tweets peaks on March 19, a few days before the global maximum of the COVID-19 tweets. On the other hand, the retweets attain their highest values later in the year 2020. This indicates that the initial wildfire was likely caused by a large number of tweets with relatively few retweets, which is quite different from the conventional idea of a single message being shared virally.

### Automated location analysis

As discussed in Sect. 2, the wildfire was initially based in the UK but affected other countries afterward. Thus, understanding the spatial distribution of the 5G-COVID misinformation tweets is necessary to understand the long-term behavior of the digital wildfire. In order to do so, we built a system to decode the self-reported locations of the Twitter users. Initially, we experimented with the tweet locations reported by Twitter, but only a small number of users enable this feature, and it is not clear whether this sample would be representative.

On the other hand, about half the tweets come from users that have a meaningful self-reported location. While it is not possible for us to determine the accuracy of the locations, we assume that there is no systematic widespread misreporting, in accordance with accepted practice in the social sciences. However, decoding the locations automatically into data that can be evaluated by country poses an additional challenge.

We solve this problem in the following way: We first count the frequency of each self-reported location string. The count shows that less than 120,000 location strings appear more than once. Therefore, it becomes possible to use the Google Geocoding API [33] which transforms the text string into a Country/State/City record. We only consider countries, and we group smaller and non-English speaking countries by continent, since they have very few English tweets. In this manner, we obtain a valid location for about half the tweets, which are then aggregated into 12 countries, groups of countries, and continents. Note that calling the Geocoding API for every individual tweet or user is possible, but prohibitively expensive, since Google charges users for each individual request.

An overview over the distribution is given in Fig. 5. As expected, the USA and the UK have by far the most tweets, followed by Nigeria. The total is three times higher than the set of tweets alone, which means that each tweet has two retweets on average in the dataset. The rate is highest in India with almost four retweets per tweet and lowest in the UK with about 1.5.

### Detailed spatiotemporal analysis

**Fig. 6:**
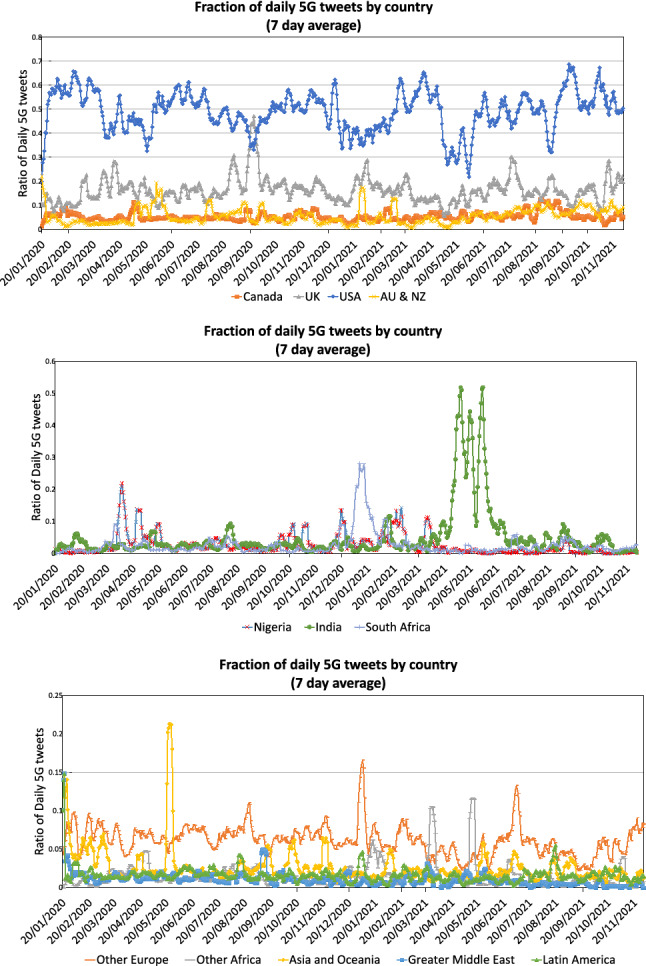
Distribution of the tweets by country over time. The given values are fractions of the total number of 5G tweets with recorded location for that day

In order to investigate the connection between the geographical and temporal distribution of misinformation tweets and real-world events, we first present the distribution of the 5G tweet set over time, divided by country or region. The results are given in Fig. 6.

In order to reduce visual clutter, we spread the data into three separate charts showing the ratio of the daily tweets. Recall from Fig. 3 that the number of tweets varies widely during the observation period, which means that the share of a country or region in early 2020 represents a far greater total number of tweets. Numbers are smoothed by taking the average over seven days.

In the top figure, we see that for Canada, the USA, the UK, Australia, and New Zealand, the ratios go up and down periodically, but a constant interest in the topic remains. On the other hand, the middle figure shows that for Nigeria, India, and South Africa, the ratios have a single high peak for a short time span, outside of which they drop to a very low level. For other world regions, which are shown in the bottom chart of Fig. 6, no consistent picture is discernible. Since most of these countries do not primarily speak English, we do not investigate them further, with the exception of the sharp peak for Asia and Oceania in May 2020. Further investigation showed this to be a result of unusual activity in Hong Kong, which we add to the observed countries in the next figure.

**Fig. 7:**
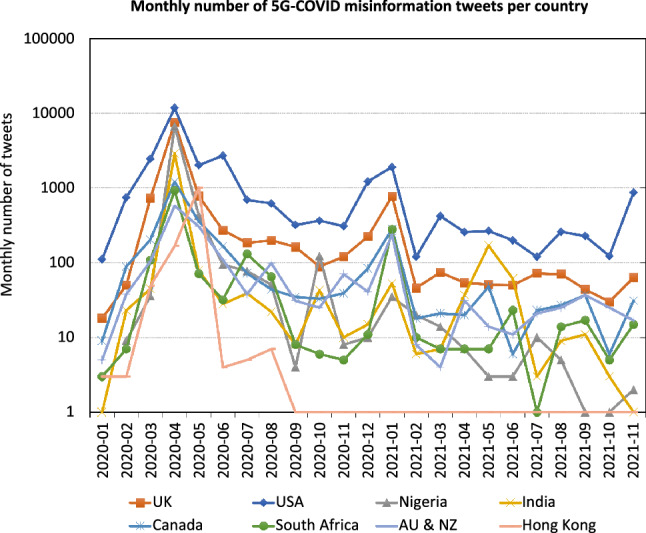
Spatiotemporal distribution of 5G-COVID misinformation

We plot the number of misinformation tweets detected by the MLP system (see Sect. 4.1) by country over time. Figure 7 shows a monthly aggregation of the number of 5G-COVID tweets. The aggregation was necessary because the daily number of misinformation tweets is quite low for some countries, and daily variance among the misinformation tweets is very high, as observed in Fig. 4. We use a logarithmic scale to adjust for the large differences between different countries and different points in time.

While almost all countries reach their maximum in April 2020, it is noteworthy that both Nigeria and the UK almost approach the US level, suggesting that these countries played a crucial role in establishing the misinformation narrative. We observe that the numbers are partially correlated, which is unsurprising since the tweets are not bound by national borders. However, the secondary peak that occurs in January 2021 coincides with a period of very high COVID-19 case numbers [43]. Furthermore, the secondary peak for India does not appear until May 2021, at the time when the country was most affected by the pandemic.

Furthermore, we observe that there is a substantial number of misinformation tweets in Hong Kong in May 2020, which quickly declines to essentially nothing afterward. (The logarithmic scale does not resolve the difference between 1 and 0.) The same is true for non-misinformation 5G tweets, but not COVID-19 tweets in general. Thus, it is likely that this decline is the result of political action, i.e., filtering all mentions of 5G, rather than a change in user behavior.

**Fig. 8:**
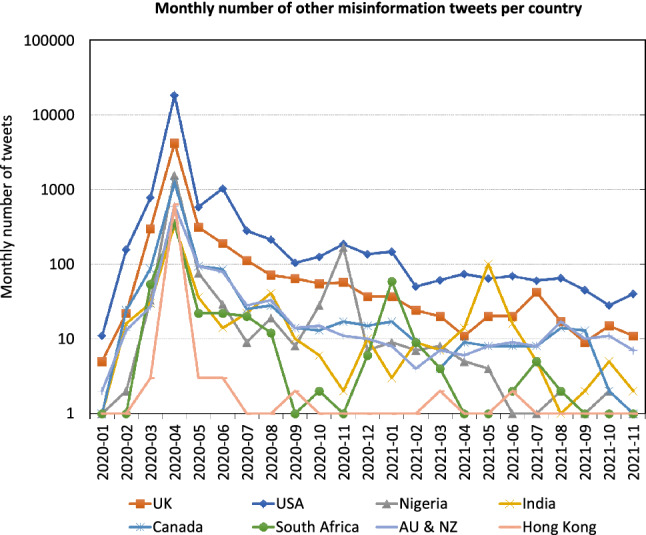
Spatiotemporal distribution of other misinformation

In Fig. 8, we show the numbers for other conspiracy theories in the same manner. Note that this only refers to tweets that mention 5G but promote conspiracy theories other than 5G-COVID misinformation. It shows a similar behavior over time in most countries (correlation of $r=0.98$). However, this is an artifact of the monthly aggregation. The correlation between the daily global numbers presented in Fig. 4 is $r=0.349$ for the absolute and $r=-0.1$ for the relative (i.e. divided by total number of tweets) number, indicating that there is no common factor that drives different types of COVID-19 misinformation.

**Fig. 9:**
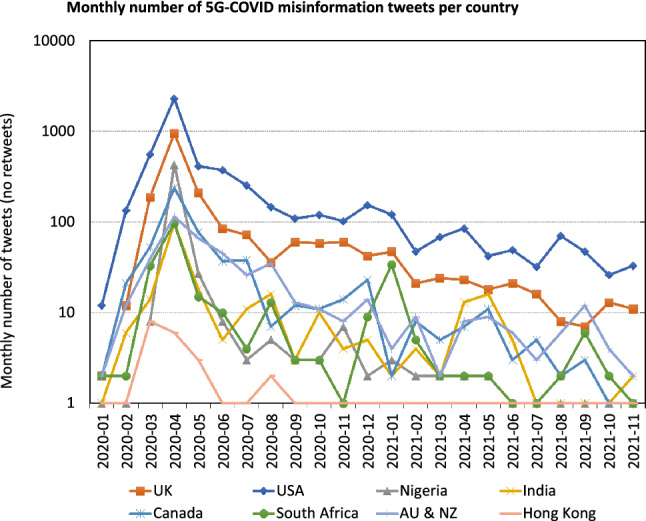
Spatiotemporal distribution of 5G-COVID misinformation excluding retweets

Finally, in Fig. 9, we consider only 5G-COVID misinformation tweets without retweets. The behavior is similar to Fig. 7, but here we observe an unusually high number of tweets in January 2021 only for South Africa. As shown in Table 3 in appendix, there was a tower attack at that time in South Africa. Thus, it is likely that the high number of 5G-COVID misinformation retweets is caused by tweet activity in South Africa, potentially by bot accounts. However, investigating these accounts is outside the scope of this study.

### Quantitative observations

We analyzed 5G-COVID and related misinformation and studied its development over time for most major English-speaking countries. The pandemic initially caused massive activity on Twitter, which slowly declined over the following 18 months, and the 5G tweets show a similar behavior. In some countries the topic has shown a consistent presence, while in others, especially India, Nigeria, and South Africa, it appeared and then disappeared quickly.

Following the cell tower attacks in early April 2020, the number of 5G tweets spiked. However, except for the case in South Africa, we do not observe a spike in misinformation tweets that predates the attacks. In this regard, the 5G-COVID digital wildfire differs from a simple digital wildfire, where an exponential spread of messages directly preceded real-world harm.

Thus, observing Twitter activity is unlikely to provide advance warning of real-world effects of a digital wildfire, at least when using the methods described here. If this is at all possible, it would require detection methods that are far more powerful (and thus computationally far more expensive) than our MPL classifier. Consequently, in the following section, we investigate other factors that might have contributed to the 5G-COVID digital wildfire.

## 5G-COVID misinformation videos

In this section, we investigate the role that video platforms such as YouTube and BitChute played in the 5G-COVID misinformation event. The analysis is predominantly qualitative, aided by processing the closed caption text that is generated automatically from videos. The investigation into the origins of the 5G-COVID misinformation event in Sect. 2.2 pointed to a series of videos which show clear links to 5G opposition that existed before January 2020.

### Opposition to 5G before COVID-19 on YouTube and other video platforms

Concerns about the safety of wireless devices have existed for a long time, and even though the scientific consensus clearly finds low-powered personal devices to be safe for human use [9], rumors to the contrary seem to persist in the many countries. The 5G standard, which was introduced in late 2018, has drawn particularly vocal criticism which in some places has delayed or stopped 5G adoption [71]. Fringe YouTube channels provide a large amount of material discussing the alleged dangers of 5G. For instance, the Swiss KlagemauerTV [45] (more than 100,000 subscribers) released more than 50 videos discussing the alleged dangers of 5G in 2018–2019 (with about 10,000 views per video). The topic was also covered by the Russian channel RT [60] in early 2019.

Furthermore, a network of activists [67] organized protests against the adoption of 5G in 2019, i.e., before the COVID-19 pandemic. In January 2020, the group repeatedly called for protests against 5G, including a “Global Day of Protest” on January 25, with the call itself appearing shortly before the first tweets linking 5G and COVID-19 appeared. The British fact-checking organization FullFact conjectures that the 5G-COVID connection grew out of this movement [30]. This conjecture is supported by our data due to the fact that many early tweets propose the *Immunosuppressor* and the *Cover-up* narratives, which are consistent with the idea that 5G is directly harmful.

### Videos promoting 5G-COVID misinformation

Among the early tweets investigated in Sect. 3.1, only one had a significant number of retweets (about 500), indicating the potential to reach a wider audience. The tweet came from an account that has almost 100,000 followers and was associated with a YouTube channel named *“Amazing Polly”* that was promoting *Mind Control Conspiracy* theories. The YouTube channel was removed in October 2020, but the videos continue to be available on BitChute and a dedicated website of the same name. While the narrative differs from other sources that oppose 5G and the conversation is not linked to similar conversations on Twitter, considering the reach of this source, it is likely that it was instrumental for the misinformation to spread in the early stages. Others content creators who had opposed 5G prior to 2020 produced their own videos soon thereafter, i.e., by the end of January 2020 [42].

Following the restrictions on public life imposed in late March 2020 by governments in Europe to slow down infection rates, a YouTube video uploaded on March 28 quickly gained popularity among anglophone conspiracy theorists. The person in the recording claims to be the former “[...] head of the largest business unit at Vodafone [...] between 2013 to 2015 [...]” and back then responsible for the implementation of IoT (Internet of Things) and 5G technologies. The Guardian [37] later was able to uncover the identity of the speaker as evangelical pastor Jonathon James who, according to The Guardian’s sources at Vodafone was hired for a sales position at Vodafone in 2014 and left the company less than a year later.

In the 30-minute recording, James presents a narrative starting with how the frequencies emitted by cell towers with 5G technology give people radiation poisoning, making the human body produce the SARS-CoV-2 virus. He claims that vaccines are dangerous and that a New World Order and Microsoft founder Bill Gates are behind a plan to “[...] pave the way for the Antichrist [...]”. On April 2, four days after its initial upload, YouTube deleted the video after it had gained substantial attention. Thereafter, it was uploaded to different YouTube channels, and later to BitChute. We found over 1,000 tweets promoting the video. On the other hand, we found only 40 tweets that mention David Icke, who is more widely known in the UK and was commonly believed to be an important factor in motivating the perpetrators of the attacks on cell phone towers [13].

There has been a significant discussion on how YouTube can lead to radicalization [5, 46]. The underlying idea is that the recommendation engine guides non-radicalized users toward increasingly radical content. However, this problem can be remedied by removing content from YouTube. On the other hand, the 5GCOVID digital wildfire was not stopped, even though many related videos were quickly removed from YouTube. Some attacks occurred several months after removal. Videos which could persuade viewers to take violent action remained on lesser known sites such as BitChute. Such sites have far fewer users, and it is unlikely that non-radicalized users find such videos by chance. However, it seems that in this case the widespread sharing of the video URL on Twitter created an alternative path to advertise persuasive videos to a significant number of viewers. This possibility should be taken into account when designing mitigation strategies for digital wildfires.

## Motivation of misinformation spreaders

So far, we have studied extensively *how* the 5G-COVID misinformation event spread, but we have not discussed *why*. In this section, we approach this question in two different lines of investigation, the first being financial and the second psychological.

### Commercial interests

On May 28, 2020, several news sources reported that London Trading Standards was targeting a British company called BIOSHIELD DISTRIBUTION LTD. for selling USB sticks as a device that “protects against harmful radiation” [16], following a recommendation by a security company that had analyzed the product [54].

The event has had little impact on Twitter, but searching the tweets for the name of the company pointed to a YouTube channel called “Connecting Consciousness” (CC) [65] that spreads different fringe ideas commonly classified as esoteric or conspiracy theories, including the idea that 5G is harmful to humans. The YouTube channel has around 50,000 viewers, with several hundred comments on each video.

The channel advertised “5G defense sticks” already in October 2019, with the CC website providing a link to a webshop, even though it claimed to be independent from the manufacturers of the device. On January 6, 2020, the channel announced delays in the fulfillment of the orders and on January 16, 2020, it hosted a presentation by the alleged inventors of the device. BIOSHIELD DISTRIBUTION LTD. was incorporated at Companies House on January 20, 2020.

Like similar products of this kind, the device seems to have been promoted at the “5G Apocalypse Event” [8] held in London in September 2019. (One of the two alleged inventors was a speaker at this event.) While the CC channel did not suggest a direct connection between 5G and COVID-19, other speakers from that event did so on January 31, 2020 [42].

The “5G Bioshield” was sold for a price of 350 USD through a professional looking website with a web shop that was still functional in July 2020. Clearly, there is a substantial commercial interest to spread misinformation for some people, and it is likely that this contributed to the digital wildfire. While many channels on YouTube pursue financial gain, it is important to distinguish between direct monetization of content, calling for donations, and the sale of questionable products. The former can easily be controlled by the video platforms. For direct monetization, the content is irrelevant as long as the videos are watched, even if viewers disagree with their content. It suffices that the viewers find it entertaining. For donations, a higher level of agreement with the content is required. However, neither of the two directly depend on a threat narrative. On the other hand, selling so-called “protective equipment” requires creating the idea of a threat. Therefore, such actors have a direct financial motivation to spread threat narratives online.

### Action and belief in conspiracy theories

While the perpetrators and thus the motivation of most of the arson incidents are not known, the perpetrators of the harassment of workers in the UK [14] and the kidnapping of technicians in Peru [1] clearly stated that the alleged dangers of 5G were the motivation for their actions, and their intentions were clearly stated in some social media channels [52]. Thus, we can assume that the 5G-COVID- related conspiracy theories played a crucial role in causing real-world harm, which is the characteristic of a *digital wildfire*.

A survey performed in early May 2020 in the UK showed that more than 20% of the respondents agree at least a little with the statement “Coronavirus is caused by 5G and is a form of radiation poisoning transmitted through radio waves,” while 1.8 % agree completely [29]. However, considering that only 0.05 % of all COVID-19 tweets even mention 5G, this seems unlikely. The methodology of the survey has been criticized since it offered only one negative and four positive answers [32]. A similar survey conducted in Norway in April 2020 with balanced positive and negative answers showed only 2.4 % agreement and 0.7 % full agreement with the statement “The 5G network has an effect on the spread of COVID-19” [26]. Furthermore, this belief was strongly correlated $(r=0.412, p \le 0.001)$ with “The magnitude of COVID-19 is exaggerated in order to persuade the world’s population to take a vaccine,” as well as other misinformation narratives.

The psychological literature has investigated the connection between paranoid thought and belief in conspiracy theories, but recent work highlighted differences between the two constructs [40]. Endorsement of conspiracy theories is not constant over time, and it depends on the situation. Conspiracy theories tend to be associated with major events, times of political instability and collective threats, such as the 9/11 terror attacks, the death of Princess Diana or the assassination of J. F. Kennedy [25, 35, 48, 64]. Having experienced stressful life events during the past six months is connected with belief in conspiracy theories [68]. Such events elicit feelings of uncertainty, and conspiracy theories can give people an explanation for a situation and its ultimate cause and hence reduce confusion. Furthermore, experimentally induced high-anxiety situations were associated with conspiracy thinking in research participants [36]. The COVID-19 pandemic is both a major and a stressful event. Moreover, it is associated with many uncertainties [55, 59] and these factors provide conspiracy theories with ideal conditions to flourish. Thus, a general willingness to endorse conspiracy theories exists, which can even cause contradictory narratives to combine into a widespread conspiracy belief, such as the 5G-COVID connection observed here, rather than counteracting each other [41].

Another study found that *“belief in 5G COVID-19 conspiracy theories was positively correlated with state anger, which in turn, was associated with a greater justification of real-life and hypothetical violence in response to an alleged link between 5G mobile technology and COVID-19”* [44], thus providing strong support for the connection between the misinformation and the arson attacks among a small group of the population.

Combining this information with the financial motivation to propose conspiracy theories or other threat narratives, the difficulties in counteracting such digital wildfires become clear. The surveys indicate that such ideas have a small group of convinced followers. While their number is apparently small, i.e., less than 1 % of the population, they have psychological reasons to believe in such threat narratives, and they are unlikely to abandon their beliefs based on statements by authorities.

Furthermore, due to the borderless nature of the internet, attempts to suppress wildfire messages proved unsuccessful, as discussed in Sect. 5. On the other hand, in Sect. 6.1 we saw that targeting BIOSHIELD DISTRIBUTION LTD. combined with demonetizing videos on YouTube appears to have been relatively successful, at least in the UK. Thus, as a general lesson from the 5G-COVID event, we conclude that eliminating the different possibilities of financially benefiting from spreading harmful misinformation is the most effective strategy for preventing or mitigating digital wildfires.

## Related work

While many studies have investigated specific rumors [24] or pieces of misinformation such as Chemtrails [72] or conspiracies [25] a significantly smaller amount of research targeted digital wildfires. Previous research on digital wildfires [74] studied the Lord McAlpine scandal [70] in which false accusation regarding child abuse were stated against the politician.

Misinformation has played a substantial role during the COVID-19 pandemic, to the extent where it has been labeled an “Infodemic” by the WHO [78] and discussed widely in the media. Several studies have investigated [39, 79] these topics.

Ahmed et. al. [4] published early results in early May 2020, shortly after the digital wildfire reached its peak. The investigation includes a graph analysis of key participant groups and essentially finds that those who participate in the discussion only believe in the narrative to a small extent. A large proportion of the participants (65.2%) merely talk about what is happening and thus indirectly contribute to its dissemination. It should be noted that the presented research is limited to the period from March 27, 2020, to April 4, 2020. This is the period in which the corresponding Twitter hashtag was trending in the UK. Moreover, Twitter is the only considered medium.

Interviewing 601 British people related to the Covid 19 and 5G conspiracy narrative, Jolley and Peterson [44] showed a positive correlation between belief in conspiracy myths and anger. Furthermore, it shows that adherents of conspiracy myths in general and adherents of the 5G-COVID conspiracy narrative in particular are more violent than those who do not believe in conspiracy myths.

Ovenseri-Ogbomo et. al. [53] administered a cross-sectional survey of 2032 participants between April 18, 2020, and May 16, 2020, focusing on beliefs regarding the 5G-COVID conspiracy narrative in sub-Saharan Africa. They found that up to 14.4% of the of the respondents in Central Africa and 7.9% on average believed in the narrative. This result complements our findings regarding the high number of misinformation tweets in Africa.

Bruns et. al. [20] analyzed more than 80,000 Facebook posts from January 1, 2020, to April 12, 2020, by retracing the dynamic of rumor dissemination through time series, qualitative, and network analysis. Compared to Twitter, Facebook represents an environment that reflects local community structures with strong ties more precisely. The resulting pockets of distinct communication act more as a Petri dish in which different branches of conspiracy myths can evolve. However, similar to our conclusion in Sect. 8, Bruns et. al. conclude that the 5G-COVID misinformation event is an amalgam of long established beliefs and narratives.

## Conclusion

The 5G-COVID misinformation event bears all the characteristics of a digital wildfire, but unlike earlier conspiracy theories which predate the widespread use of social media, it was possible to trace the 5G-COVID misinformation to its very beginning on Twitter, giving us deep insights into the origins of the event. Based on that, our analysis produced four key findings:

1. The wildfire builds on existing ideas that are interpreted in the light of the new situation.
2. The wildfire depends on an interplay between different social media platforms. Twitter allows ideas to spread among a relatively small group, while thematically focused video channels reach a relatively large audience. On the other hand, news websites played no significant role except by reporting on real-world harm.
3. The existence of mutually exclusive narratives has strengthened the 5G-COVID misinformation event rather than weakening it.
4. A pure Twitter analysis underestimates the effect of videos with commercial interests and large numbers of viewers.

We believe that for mitigating future digital wildfires, it is important to take all four observations into account. However, doing so is technologically challenging, especially w.r.t. the second finding. In previous work, the concept of information cascade has often been equated with a single tweet and its retweets [10]. However, for the 5G-COVID misinformation event we observe that misinformation did not only spread through a large number of tweets with comparatively small influence but also in parallel on other social media channels. This ties in with the third observation. Not only did the information cascade consist of a multitude of individual messages, but the messages also contained conflicting narratives. One could argue that this should be classified as a set of individual information cascades instead, but all these messages strengthened the core idea of the digital wildfire, i.e., that 5G and COVID-19 are somehow linked.

There is a broad consensus in many countries that digital wildfires constitute a threat to society [27] and that measures should be taken to counteract them. However, there is a considerable disagreement on which measures are appropriate to do so. It is important to remember that although the events can be shocking, the property damage was moderate and so far, no lives were lost. Thus, censorship, which apparently happened in Hong Kong, would be grossly disproportionate. Instead, we formulate the following four policy recommendations:

1. Targeting content, e.g., by removing offending videos, was not particularly successful, since alternatives to YouTube were readily available and Twitter messages promoted videos on alternative sites. Furthermore, removing content often constitutes, or is perceived as, a form of censorship, which can erode trust in authorities. Thus, it may be preferable to retain content and instead prevent it from being advertised widely (e.g., via the recommendation engine on YouTube).
2. Targeting financial motivations of misinformation spreaders is far more feasible and has fewer political risks than content removal. Both demonetization of content and investigation of fraudulent products can be advisable.
3. While the prediction of digital wildfires appears to be extremely difficult to achieve with current technology, ex-post observation of digital wildfires can be valuable because the same misinformation may cause harm more than once. Several 5G-COVID-related attacks happened in 2021, several months after the first wave of events.
4. Finally, it is important to share results obtained in this manner internationally, since a wildfire is more likely to reappear in a country that was so far unaffected. This is especially true for counties that have little contact with the original place in which the wildfire occurs, as evidenced by the kidnapping of technicians in Peru.

While the effects of 5G-COVID were not as devastating as those of other misinformation, it is important to consider that 5G is envisioned to provide extreme reliability for automotive and industrial applications. Therefore, damage to the infrastructure could have much more serious consequences in the future [17].

Despite the difficulties, the analysis of social media provides the best chance for detecting potential digital wildfires while they unfold. In future work, we will investigate the use of more advanced natural language processing methods to sharpen the automated analysis, as well as enable the investigation of different misinformation narratives at the same time.

**Table 2:** The collection of 5G-Corona news articles found in the GDELT database for the period from February 20 to 27 (identical titles represent articles that are essentially copies of other articles)

Date	Supports 5G-Corona conspiracy	Number of referred events	Publisher	Title
2020-02-20	No	1	lightreading.com	Coronavirus cuts into 5G standards work
2020-02-20	No	12	reuters.com	Huawei says no impact on 5G supply from coronavirus
2020-02-20	No	12	investing.com	Huawei says no impact on 5G supply from coronavirus
2020-02-20	No	11	marketscreener.com	Huawei says no impact on 5G supply from coronavirus
2020-02-20	No	14	reuters.com	Huawei says no impact on 5G supply from coronavirus
2020-02-20	No	11	oann.com	*# URL not found*
2020-02-20	No	3	cnn.com	Apple needs a 5G iPhone now more than ever
2020-02-20	**YES**	1	veteranstoday.com	UPDATE On Coronavirus, 5G, ELF, Apirin, Mismanagement - the Perfect Storm
2020-02-20	No	11	phonearena.com	Production of Huawei’s 5G networking equipment unaffected by the coronavirus
2020-02-20	No	11	cnbc.com	UPDATE 1-Huawei says no impact on 5G supply from coronavirus
2020-02-21	No	13	abs-cbn.com	Huawei says no impact on 5G supply from coronavirus
2020-02-21	No	11	indiatimes.com	Huawei says no impact on 5G supply from coronavirus
2020-02-21	No	6	reuters.com	China’s ambitious 5G push heading into slow lane due to coronavirus disruptions
2020-02-21	No	6	reuters.com	China’s ambitious 5G push heading into slow lane due to coronavirus disruptions
2020-02-21	No	6	reuters.com	China’s ambitious 5G push heading into slow lane due to coronavirus disruptions
2020-02-21	No	6	channelnewsasia.com	China’s ambitious 5G push heading into slow lane due to coronavirus disruptions
2020-02-21	No	6	oann.com	China’s ambitious 5G push heading into slow lane due to coronavirus disruptions
2020-02-21	No	6	reuters.com	China’s ambitious 5G push heading into slow lane due to coronavirus disruptions
2020-02-21	No	4	businesstimes.com.sg	China’s ambitious 5G push heading into slow lane due to coronavirus disruptions
2020-02-21	No	4	msn.com	China’s ambitious 5G push heading into slow lane due to coronavirus disruptions
2020-02-21	No	4	indiatimes.com	China’s ambitious 5G push heading into slow lane due to coronavirus disruptions
2020-02-21	No	4	indiatimes.com	China’s ambitious 5G push heading into slow lane due to coronavirus disruptions
2020-02-21	No	6	marketscreener.com	China’s ambitious 5G push heading into slow lane due to coronavirus disruptions
2020-02-21	No	2	theepochtimes.com	China’s ambitious 5G push heading into slow lane due to coronavirus disruptions
2020-02-21	No	1	marketwatch.com	Opinion: The 5G rollout is already behind, and coronavirus could slow it even more
2020-02-21	No	6	msn.com	China’s ambitious 5G push heading into slow lane due to coronavirus disruptions
2020-02-21	No	2	ndtv.com	China’s ambitious 5G push heading into slow lane due to coronavirus disruptions
2020-02-21	No	1	lightreading.com	*# URL not found*
2020-02-22	*Nothing for the date*			
2020-02-23	No	1	nyoooz.com	China’s ambitious 5G push slows due to coronavirus disruptions
2020-02-24	No	7	sputniknews.com	India Gets ’First 5G Smartphone’ From China Amid Delayed Trials Due to Coronavirus
2020-02-24	No	4	computerweekly.com	Coronavirus, global economic slowdown to cap 5G smartphone sales in 2020
2020-02-25	No	3	zdnet.com	Jabil cuts outlook as coronavirus curbs manufacturing for tech giants, 5G ecosystem
2020-02-26	*Nothing for the date*			
2020-02-27	*Nothing for the date*			

**Table 3:** A collection of news articles we used to keep track of worldwide real-world consequences linked to the 5G-Corona misinformation event (arson attacks and harassment of telecommunication technicians)

Date	#e	#m	Country	Location	Media/Source	Title	Journalist/editor	Link
2020-01-02	1		DE	Bonn	General Anzeiger	Polizei vermutet Brandstiftung nach Feuer an ...	Silke Elbern	3p3rrtL
2020-03-27	1	14	NZ	Waiharara	MSN	Suspected cell tower arsons prompt call for witnesses	Liu Chen	2TsBzRl
2020-04-02	1	30	IE	Belfast	New York Times	Burning Cell Towers, Out of Baseless Fear ...	Adam Satariano et al.	34v92g0
2020-04-02	1		UK	Birmingham	The Sun	HOLY SMOKE Idiots “BURNING 5G masts” ...	Holly Christodoulou	34sYnCH
2020-04-03	2	30	UK	Liverpool	Independent	Coronavirus 5G conspiracy theory ...	Adam Satariano et al.	3fS0VPX
2020-04-04	1	4	NL	Beesd	Telegraaf	Wéér incident bij mast: verzet 5G wordt militant	Martin Nuver	3fy4vQm
2020-04-05	1	14	NZ	Manurewa	MSN	Suspected cell tower arsons prompt ...	Liu Chen	3vtShhc
2020-04-05	1		NL	Liessel	Omroepbrabant	Leuzen tegen 5G bij brandende telefoonmast in Liessel	Femke de Jong	34Hcrc1
2020-04-05	1		NL	Rotterdam	Telegraaf	Wéér incident bij mast: verzet 5G wordt militant	Martin Nuver	3wNbGdr
2020-04-05	1		NL	Deurne	DMG	Brand in GSM-mast Liessel waarschijnlijk ...	Ivo Boudewijns	2R6S1G6
2020-04-09	1		NL	Nuenen	Omroepbrabant	Brand in telefoonmast in Nuenen, mogelijk aangestoken	Peter de Bekker	3p3vF4Y
2020-04-10	1		NL	Groningen	Telegraaf	Wéér incident bij mast: verzet 5G wordt militant	Martin Nuver	34HcyEt
2020-04-10	1		NL	Oudenbosch	NOS	Waarom worden door heel ...?	ANP	2RTf2wx
2020-04-11	1	20	UK	Birmingham	Telegraph	Birmingham Nightingale phone mast ...	Matthew Field	3vKNQip
2020-04-11	1		UK	West Yorkshire	Telegraph	Birmingham Nightingale phone ...	Matthew Field	3i2ubGE
2020-04-11	1	Several	CY	Limassol	Ars Technica	How a 5G coronavirus ...	Nic Fildes et al.	3fuCjOc
2020-04-11	0	22	UK	Several locations	Businessinsider	Vandals set 50 cellphone masts in the ...	Isobel Asher Hamilton	3cl4e1p
2020-04-11	1		NL	Veldhoven in Brabant	NOS	Waarom worden door heel Nederland zendmasten ...?	ANP	3c4jdfP
2020-04-11	2		IE	Co Donegal	Irish Times	Gardaí suspect masts set on fire deliberately in Co Donegal	Stephen Maguire	3fwdPV5
2020-04-13	1	Several	NL	Amsterdam (Almere)	Ars Technica	How a 5G coronavirus conspiracy spread across Europe	Nic Fildes et al.	3i16NJq
2020-04-14	1	20	UK	Dagenham, Essex	BBC	Coronavirus: 20 suspected phone mast attacks over Easter	Leo Kelion	2SFdMgE
2020-04-17	1	77	UK	Huddersfield	Wired	The 5G coronavirus conspiracy ...	James Tamperton	34uiP6c
2020-04-19	1		DE	Bonn	General Anzeiger	Gibt es in Bonn eine Brandanschlagserie auf Funkmasten?	Ayla Jacob	2RSoNeg
2020-04-22	1		DE	Wilhelmshaven	Police press	POL-WHV: 30-Jähriger durchtrennt ...		3fWykcb
2020-04-28	1	Several	NZ	Papatoetoe	MSN	Suspected cell tower arsons prompt call for witnesses	Liu Chen	3fVCuBc
2020-05-01	1		CA	Montreal	CTV News	Cell tower set on fire north of Montreal	Katelyn Thomas	34qTkCz
2020-05-04	1		CA	Piedmont	CTV News	Two more cell towers went up in flames north of Montreal	Katelyn Thomas	3uxRJWf
2020-05-04	1		CA	Prévost	CTV News	Two more cell towers went up in flames north of Montreal	Katelyn Thomas	3c2tEQV
2020-05-05	1		CA	Laval	CTV News	Another cell tower in Quebec - the fourth ...	Katelyn Thomas	3uBWzSh
2020-05-12	1		NZ	Māngere	MSN	Suspected cell tower arsons prompt call for witnesses	Liu Chen	3c1Tftr
2020-05-15	1		NZ	Ōtāhuhu	MSN	Suspected cell tower arsons prompt call for witnesses	Liu Chen	3p8r80X
2020-05-15	1		NZ	Favona	MSN	Suspected cell tower arsons prompt call for witnesses	Liu Chen	3i7ZT5n
2020-05-18	1		AU	Melbourne	news.com.au	Cranbourne West phone tower fire: Counter-terrorism ...	Jack Paynter	3fwCQzh
2020-08-06	3	20	NZ	South Auckland	TVNZ	Cell phone towers burned in latest ’senseless’ arson ...	Luke Appleby	3vC0TCG
2020-06-10	8		PE	Provinz Acobamba	RPP	Huancavelica: Secuestran a 8 ingenieros que arreglaban ...	Redacción	2RTRoA2
2020-07-01	1		CY	Limassol	KNEWS	More 5G targets torched overnight	unknown journalist	3uBWSfT
2020-08-10	1		UK	Essex	Essex Live	Police fear Chelmsford 5G mast ’will fall over’ ...	unknown journalist	3vwXrZO
2020-09-10	1	90	UK	Bradford	Daily Mail	Arsonists destroy another 5G mast fueled by false ...	Clare McCarthy	3wIETG6
2020-09-23	1		UK	Bradford	Telegraph & Argus	Police appeal for information as 5G mast set on ...	unknown journalist	3vzMw1t
2020-11-05	1		UK	Bierley	Telegraph & Argus	Firefighters called to 5G mast blaze in Bierley	Felicity Macnamara	3yOPUYk
2021-01-05	4		ZA	Durban, KwaZulu-Natal	Daily Maverick	KZN cellphone towers torched as 5G ...	Rebecca Pitt	2RTfM4N
2021-01-31	3		UK	Chelmsford, Essex	Telegraph	Conspiracy theorists could be behind ...	Martin Evans	3p2bQLc
2021-03-31	1		CA	Scarborough	Toronto Citynews	Police say cell phone tower fire in ...	“News Staff”	3p3thuF
2021-10-09	1		FR	Villié-Morgon	Le Parisien	Rhône : plongée dans le couvent des croisés de la 5G	Vincent Mongaillard	3dAdrmI

Column #e contains the number of events with an exact location mentioned in the article, #m all mentioned events in the article. Although the original table was much larger, this selection contains only those articles with precise location information. Links start with https://bit.ly/

## Appendix

**Search Keywords** list of search English keywords for obtaining the initial set of COVID tweets was as follows: *#corona*, *corona*, *covidiot*, *#covidiot*, *#coronaoutbreak*, *#coronarvirus*, *#coronavirus*, *#coronavirusde*, *#coronavirusoutbreak*, *#covid*, *#covid19*, *#covid2019*, *#covid_19*, *#covid-19*, *#wuhan*, *#wuhancoronavirus*, *#wuhancoronovirus*, *#wuhanvirus*, *coronarvirus*, *coronavirus*, *coronavirusde*, *coronavírus*, *covid*, *covid-19*, *covid19*, *covid2019*, *covid_19*, *covid-19*, *epidemic*, *pandemic*, *quarantine*, *quarantined*, *wuhan*.

The search was performed concurrently with searches in other languages. However, only English tweets were included in the dataset.
